# Prognosis of ischemic stroke predicted by machine learning based on multi-modal MRI radiomics

**DOI:** 10.3389/fpsyt.2022.1105496

**Published:** 2023-01-09

**Authors:** Huan Yu, Zhenwei Wang, Yiqing Sun, Wenwei Bo, Kai Duan, Chunhua Song, Yi Hu, Jie Zhou, Zizhang Mu, Ning Wu

**Affiliations:** ^1^Department of Radiology, Liangxiang Hospital, Beijing, China; ^2^Department of Neurology, Liangxiang Hospital, Beijing, China; ^3^Department of Medical Imaging, Yanjing Medical College, Capital Medical University, Beijing, China

**Keywords:** diffusion-weighted imaging, radiomics, machine learning, ischemic stroke, magnetic resonance imaging

## Abstract

**Objective:**

Increased risk of stroke is highly associated with psychiatric disorders. We aimed to conduct the machine learning model based on multi-modal magnetic resonance imaging (MRI) radiomics predicting the prognosis of ischemic stroke.

**Methods:**

This study retrospectively analyzed 148 patients with acute ischemic stroke due to anterior circulation artery occlusion. Based on the modified Rankin Scale (mRS) score, patients were divided into good (mRS ≤ 2) and poor (mRS > 2) outcome groups. Segmentation of the infarct region was performed by manually outlining a mask of the lesion on diffusion-weighted images (DWI) using MRIcron software. The apparent diffusion coefficient (ADC), fluid decay inversion recoverage (FLAIR), susceptibility weighted imaging (SWI) and T1-weighted (T1w) images were aligned to the DWI images and the radiomic features within the lesion area were extracted for each image modality. The calculations were done using pyradiomics software and a total of 4,744 stroke-related imaging features were automatically calculated. Next, feature selection based on recursive feature elimination was used for each modality and three radiomic features were extracted from each modality plus one feature from the lesion mask, for a total of 16 radiomic features. At last, five machine learning (ML) models were trained and tested to predict stroke prognosis, calculate the received operating characteristic (ROC) curves and other parameters, evaluate the performance of the models and validate their predictive efficacy by five-fold cross-validation.

**Results:**

Sixteen radiomic features were selected to construct the ML models for prognostic classification. By five-fold cross-validation, light gradient boosting machine (LightGBM) model-based muti-modal MRI radiomic features performed best in binary prognostic classification with accuracy of 0.831, sensitivity of 0.739, specificity of 0.902, F1-score of 0.788 and an area under the curve (AUC) of 0.902.

**Conclusion:**

The ML models based on muti-modal MRI radiomics are of high value for predicting clinical outcomes in acute stroke patients.

## 1. Introduction

Psychiatric comorbidities, such as depression (1), anxiety (2) and dementia (3), are the frequent consequences of stroke, which is one of the leading causes of disability and death worldwide (4). The psychiatric disorders make the prognosis of stroke complicated, and the prognosis varies greatly depending on the time of consultation and treatment, which lead to a challenge in deciding of “when to treat” and “how to treat” during rehabilitation of stroke patients (5). The accurate prediction of rehabilitation outcomes will do great help to propose the appropriate treatment strategies and rehabilitation goals based on each patient’s condition (6).

The combination of the multi-modal magnetic resonance imaging (MRI) techniques provides a powerful tool for stroke diagnosis. Mitra et al. (7) used the information from multimodal [T1-weighted, T2-weighted, fluid attenuated inversion recovery (FLAIR), and apparent diffusion coefficient (ADC)] MRI images to extract areas with high likelihood of being classified as stroke lesions. Radiomics is an emerging approach that combines imaging and artificial intelligence to extract quantitative features from images in high throughput. Zhang et al. (5) developed the machine learning model-based diffusion weighed imaging (DWI)/ADC radiomic features to classify ischemic stroke onset time. Quan et al. (8) constructed the unfavorable outcome model based on the radiomic feature extracted from FLAIR and ADC image. Moreover, susceptibility weighted imaging (SWI), reflecting the oxygen extraction fraction of brain tissues, has been demonstrated as a useful predictor of early infarct size and early-stage clinical prognosis in acute ischemic stroke (9).

In this study, we constructed five machine learning (ML) models that aimed to predict the prognosis of ischemic stroke patients based on muti-modal MRI radiomics. In addition, we assessed predictive value of the models for ischemic stroke treatment decision-making.

## 2. Materials and methods

### 2.1. Participants

**FIGURE 1:**
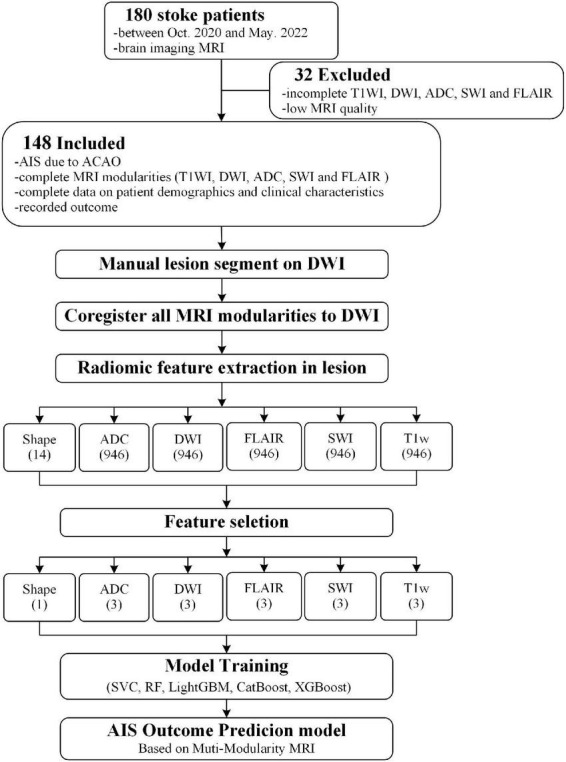
Flow chart of participant selection and analysis. DWI, diffusion-weighted imaging; ADC, apparent diffusion coefficient; FLAIR, fluid attenuated inversion recovery; SWI, susceptibility weighted imaging; ML, machine learning; SVM, support vector machine, RF, random forest; LightGBM, light gradient boosting; CatBoost, category boosting, and XGBoost, eXtreme gradient boosting.

This study was a retrospective analysis of 180 patients diagnosed with acute ischemic stroke at Liangxiang Hospital (Beijing, China) from October 2020 to May 2022, of which 148 were included in analysis (Figure 1). The inclusion criteria were: (1) acute ischemic stroke due to anterior circulation artery occlusion; (2) MRI completed within 48 h of admission; (3) complete set of MRI sequences; (4) complete data on demographics and clinical characteristics; and (5) signed informed consent. The exclusion criteria were: (1) cerebral hemorrhage; (2) traumatic brain injury; (3) previous neurological or psychiatric disease; and (4) significant artifacts in MRI data. This study was approved by the ethics committee of Liangxiang Hospital (approval number 2016126).

Demographic characteristics as well as the clinical and imaging data were collected under the permission of patients. The National Institutes of Health Stroke Scale (NIHSS) score was collected to evaluate the degree of neurological deficit in stroke patients, which represented the level of consciousness, eye movements, integrity of visual fields, facial movements, arm and leg muscle strength, sensation, coordination, language, speech and neglect (10). Arranging from 0 to 42, the higher the NIHSS score, the more severe the neurological impairment: score 0 was normal neurological function, 1 to 4 was mild stroke, 5 to 15 was moderate stroke, 16 to 20 was moderate-severe stroke and 21 to 42 was severe stroke.

The modified Rankin Scale (mRS) score was used as a prognostic judgment index, with good prognosis defined as mRS scores of 0, 1, and 2, and poor prognosis defined as mRS scores of 3, 4, and 5. Of the 148 patients included in the analysis, 83 were in the good prognosis group and 65 were in the poor prognosis group.

### 2.2. MR image acquisition

MRI scans were performed within 3 days of stroke onset, using a Magnetom Skyra 3.0T MRI scanner (Siemens, Germany) with a 20-channel phased-array head coil.

All participants underwent the following scans:

(1) T1-weighted image scan. Scan parameters: T1w sequence, repetition time (TR) = 2,000 ms, inversion time (TI) = 900 ms, echo time (TE) = 8.8 ms, matrix = 209 × 256, field of view (FOV) = 220 mm^2^ × 196 mm^2^, thickness = 5 mm. number of layers = 24 layers, and parallel imaging factor = 2.

(2) Cerebrospinal fluid suppression image. Scan parameters: T2-FLAIR sequence, TR = 6,000 ms, TI = 2,028 ms, TE = 72 ms, matrix = 320 × 261, FOV = 220 mm^2^ × 196 mm^2^, thickness = 5 mm, number of layers = 24 layers, and parallel imaging factor = 2.

(3) SWI imaging scan sequence. Scan parameters: 3D-GRE sequence, TR = 27 ms, TE = 20 ms, flip angle (FA) = 15°, matrix = 256 × 256, FOV = 220 mm^2^ × 196 mm^2^, layer thickness = 2.5 mm, number of layers = 44, repetition number = 1, fat suppression on, and parallel imaging factor = 2.

(4) DWI imaging scan sequence. Scan parameters: EPI-Resolve sequence, b-value b = 1,000 and 0 scan, TR = 500 ms, TE1 = 63 ms, TE2 = 103 ms, FA = 180°, matrix = 160 × 160, FOV = 220 mm^2^ × 220 mm^2^, layer thickness = 5 mm, number of layers = 24, fat suppression on, and parallel imaging factor = 2.

### 2.3. Image processing and segmentation

Image analysis was performed independently by two MR diagnosticians blinded to the groups. The region of interest (ROI) of acute ischemic lesions was manually outlined layer by layer on the DWI images using MRIcron software.^1^

**FIGURE 2:**
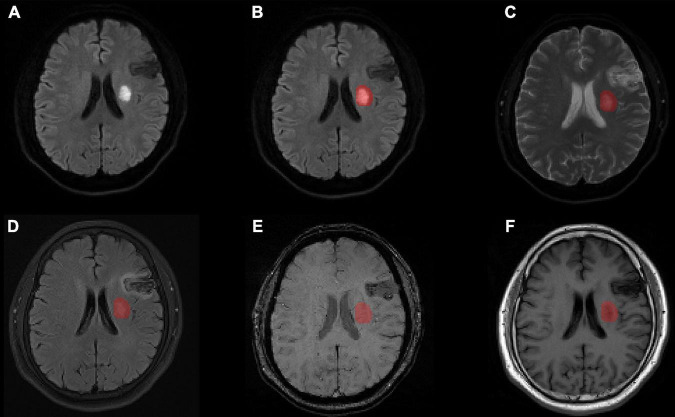
Lesion segmentation results for one patient with manual outlining of the lesion using MRIcron on **(A)** the original DWI image, **(B)** the lesion superimposed onto the DWI image, **(C)** the lesion superimposed onto the ADC image, **(D)** the lesion superimposed onto the FLAIR image aligned to the DWI image, **(E)** the lesion superimposed onto the SWI image aligned to the DWI image, and **(F)** the lesion superimposed onto the T1w image aligned to the DWI image. MRI, magnetic resonance imaging; ADC, apparent diffusion coefficient; DWI, diffusion-weighted imaging; FLAIR, fluid attenuated inversion recovery; SWI, susceptibility weighted imaging; T1w, T1-weighted.

For each patient, data including all modalities (ADC, FLAIR, SWI, T1w) were aligned to the DWI images using SPM12 software^2^ so that the outlined lesions could be directly used for texture feature extraction in the different modal images (Figure 2).

### 2.4. Radiomic feature extraction

For each patient, the MRI data of the five modalities was analyzed by pyradiomics software^3^ for radiomiscs feature extraction using the recommended settings and steps for MRI data: (1) Images were resampled to 3 mm^2^ × 3 mm^2^ × 3 mm. (2) DWI, FLAIR, SWI, and T1w images are weighted images and thus needed to be numerically standardization. A scale of 100 was used and the binwith was set to 5. Since ADC images are quantitative, no numerical standardization was done and binwith was set to 20. The above settings ensured that the total number of bins is between 16 and 128. (3) Texture feature extraction was performed on the original images and filtered images, where the filter consisted of Laplacian of Gaussian filter based on sigma = 3 and 5 mm, edge enhancement filter, and 8 wavelet transforms (combination of high pass and low pass in three dimensions). (4) Finally, the texture features of radiomics were extracted, including 18 first order, 22 glcm, 16 glrlm, 16 glszm, and 14 gldm features. Thus, a total of 946 features were extracted per image. The shape features of 14 lesion regions were also extracted.

### 2.5. Feature selection and model training based on ML

The scikit-learn package^4^ was used for feature selection of the MRI data of the five modalities. The recursive feature elimination (RFE) feature extraction method was used and only the three best features were retained for each modality. For lesion shape features, we used the same method to retain the one best feature. Finally, a total of 16 image features were retained and used to train the ML model with the scikit-learn tool. A total of five methods, including Support Vector Machine (SVM) Classifier, Random Forest (RF) Classifier, Light Gradient Boosting Machine (LightGBM) Classifier, Category Boosting (CatBoost) Classifier, and eXtreme Gradient Boosting (XGBoost) Classifier, were used to build the models. Model performance was evaluated by a five-fold stratified cross-validation process. The evaluation metrics included accuracy, precision, recall, F1 score, receiver operating characteristic (ROC) curve, area under the curve (AUC), precision recall curve.

### 2.6. Statistical analysis

Statistical analysis was performed using SPSS 21.0 software. The independent samples *t*-test was used to compare the measurement data. Results with *p* < 0.05 were considered to be statistically significant differences.

## 3. Results

### 3.1. Demographic characteristics of patients

Of the 148 patients, 83 (56.1%) had a good prognosis and 65 (43.9%) had a poor prognosis. The training set comprised 104 patients and the remaining 44 were used to test the ML model. For the overall sample, the mean age was 64.29 years and the mean NIHSS score was 5.59. These two variables differed significantly between groups (*p* < 0.05). No significant differences were found between patient groups for other baseline clinical characteristics (all *p* > 0.05, Table 1).

**TABLE 1:** Baseline demographic and clinical characteristics.

Characteristics	Good prognosis (mRS ≤ 2) (*n* = 83)	Poor prognosis (mRS > 2) (*n* = 65)	*t*/χ^2^	*P*-value
Age, year, mean ± SD	59.21 ± 10.94	70.80 ± 10.92	–6.404	0.001
Male, *n* (%)	62 (74.70)	38 (58.46)	4.386	0.036
NIHSS score, mean ± SD	2.33 ± 1.75	9.75 ± 5.65	–10.222	0.001
Hypertension, *n* (%)	61 (73.49)	56 (86.15)	3.528	0.060
Diabetes, *n* (%)	43 (51.81)	27 (41.54)	1.542	0.214
History of coronary heart disease, *n* (%)	4 (4.82)	13 (20.00)	8.263	0.004
History of atrial fibrillation, *n* (%)	3 (3.61)	11 (16.92)	7.539	0.006
Smoking, *n* (%)	55 (66.27)	31 (47.69)	5.166	0.023
Drinking, *n* (%)	45 (54.22)	22 (33.85)	6.105	0.013
Complications, *n* (%)	1 (1.20)	36 (55.38)	57.069	0.001

NIHSS, National Institutes of Health Stroke Scale; mRS, modified Rankin Scale.

### 3.2. Radiomic feature extraction and selection

Three best radiomic features for each MRI modality (DWI, ADC, FLAIR, SWI and T1w) and one best feature for lesion shape were selected as features in the ML models. The detailed information about the features is presented in Table 2.

**TABLE 2:** Results of feature selection for each MRI modality.

	Feature 1	Feature 2	Feature 3
Shape	MeshVolume		
DWI	log-sigma-3-0-mm-3D_glrlm_ LowGrayLevelRunEmphasis	log-sigma-3-0-mm-3D_glrlm_ ShortRunLowGrayLevelEmphasis	wavelet-LLH_glcm_Idn
ADC	log-sigma-5-0-mm-3D_firstorder_Maximum	log-sigma-5-0-mm-3D_firstorder_TotalEnergy	wavelet-HLL_gldm_ LargeDependenceHighGrayLevelEmphasis
FLAIR	original_firstorder_90Percentile	log-sigma-3-0-mm-3D_glszm_LargeAreaEmphasis	log-sigma-3-0-mm-3D_glszm_ LargeAreaHighGrayLevelEmphasis
SWI	log-sigma-5-0-mm-3D_glcm_Idmn	wavelet-HHH_glcm_Imc1	wavelet-HHH_glcm_Imc2
T1w	original_glszm_ZoneVariance	log-sigma-5-0-mm-3D_glszm_ LargeAreaLowGrayLevelEmphasis	wavelet-LLL_glcm_Imc1

ADC, apparent diffusion coefficient; DWI, diffusion-weighted imaging; FLAIR, fluid attenuated inversion recovery; SWI, susceptibility weighted imaging; T1w, T1-weighted.

### 3.3. Training and evaluation of ML prediction models

**FIGURE 3:**
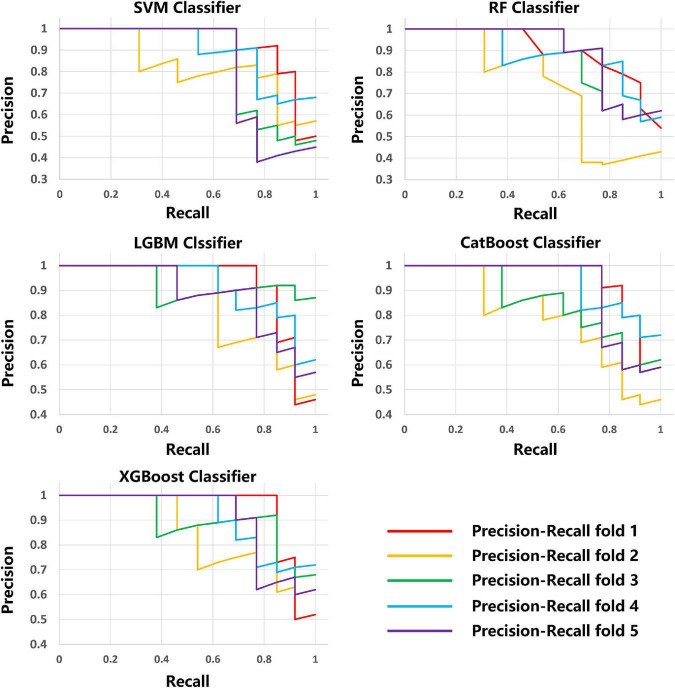
Precision-recall curves of the five models on five-fold cross-validation.

**FIGURE 4:**
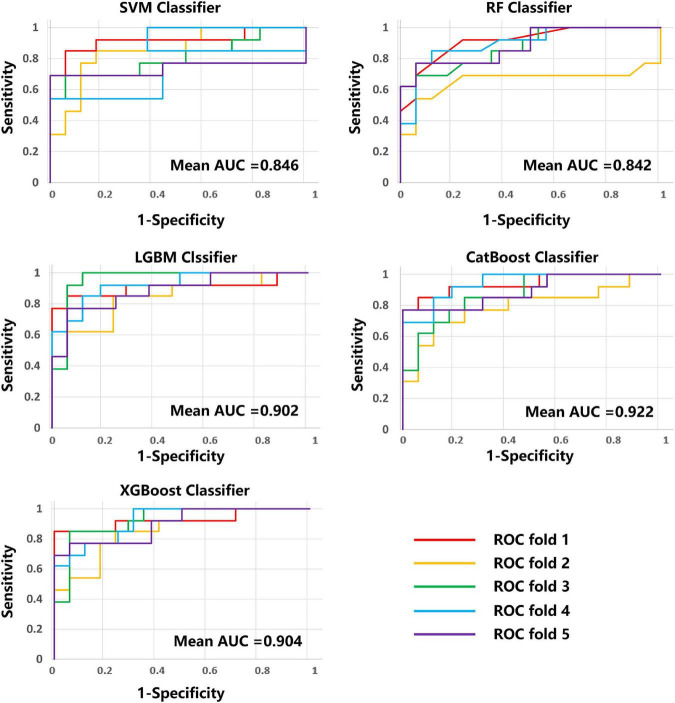
ROC curves and AUC values of the five models on five-fold cross-validation. ROC, received operating characteristic; AUC, area under the curve.

The average results obtained for the test set using the different classification models after five-fold cross-validation are as follows: SVM model with 79% accuracy, RF model with 82% accuracy, LightGBM model with 83% accuracy, CatBoost model with 81% accuracy, and XGBoost model with 80% accuracy. The full model evaluation results are shown in Table 3 and Figures 3, 4.

**TABLE 3:** Results of different models for the test set (average results of five-fold).

Model	Accuracy	Sensitivity	Specificity	Precision	Recall	F1-score
SVM	0.791	0.631	0.915	0.858	0.631	0.722
RF	0.818	0.723	0.891	0.838	0.723	0.773
LightGBM	0.831	0.739	0.902	0.875	0.739	0.787
CatBoost	0.812	0.662	0.928	0.876	0.662	0.748
XGBoost	0.804	0.708	0.878	0.833	0.708	0.753

SVM, support vector machine Classifier; RF, random forest; LightGBM, light gradient boosting machine; XGB, extreme gradient boosting; F1-score = 2 × (precision × recall)/(precision + recall).

## 4. Discussion

Early and accurate determination of disease progression may be important for new stroke patients, allowing timely targeted treatment and effective improvement. To predict the prognosis of AIS early and accurately, this paper investigated five ML models based on multi-modal (T1w, ADC, DWI, FLAIR, and SWI) MRI radiomic features to predict AIS prognosis. The results showed that LightGBM model performed best in binary prognostic classification with accuracy of 0.831, sensitivity of 0.739, specificity of 0.902, F1-score of 0.788 and an area under the curve (AUC) of 0.902.

At present, most studies on the prognosis of ischemic stroke have used retrospective cohort studies to perform traditional statistical analysis of stroke prognosis models using Cox regression and logistic regression (11–13). Previous studies have failed to make full use of MRI data, resulting in low prediction accuracy (14–16). Several studies support that ML can predict stroke prognosis more accurately (17–19). Wang et al. (20) showed that, despite variability, current ML-based prognosis prediction of stroke patients has great potential. Qu et al. (21) used ML of retinal images to assess risk in 771 patients with ischemic and hemorrhagic stroke, achieving sensitivity and specificity of ischemic stroke risk assessment values of 91.0 and 94.8%, respectively. The area under the ROC curve for ischemic stroke was 0.929. Cui et al. (22) applied ML to develop and validate the incidence and severity of acute ischemic stroke in 1,100 patients. The combination of ML methods (e.g., complex neural networks) with imaging omics seems particularly promising, especially for the identification and segmentation of small lesions (23–25). Macciocchi et al. (26) performed a 3 month systematic evaluation of ischemic stroke and concluded that characteristics, such as age, previous stroke, initial neurological deficit, and lesion location, were highly correlated with functional outcome. The current results are consistent with those of previous studies, suggesting that imaging histology scores, hemorrhage, age, and NIHSS at 24 h are independent indicators of clinical outcome in patients with ischemic stroke. By combining these independent risk factors to generate a new imaging histology line graph, several studies have reported an association of DWI-derived ADC changes with functional outcome in ischemic stroke (27). A previous study reported that DWI had a 90% probability of identifying a lesion within 3 h prior to symptom onset (28). The present study suggested that radiomic features based on multi-modal MRI could predict clinical outcomes in acute stroke patients with accuracy of 0.831.

This study has provided new clues for predicting the prognosis of AIS and demonstrated the ability of multi-modal based radiomics to accurately predict the clinical functional outcome of AIS, contributing to the prevention of post-stroke psychiatric diseases. However, this study still has several limitations. First, this was a retrospective study with selection bias. Studies using larger samples are needed to further validate the predictive efficacy of the model. Second, this study did not differentiate the etiology and site of stroke, and manual outlining of ROI was affected by individual subjective factors. These clinical and imaging data should be considered in further study in the next step.

## Data availability statement

The raw data supporting the conclusions of this article will be made available by the authors, without undue reservation.

## Ethics statement

This study was approved by the Ethics Committee of Liangxiang Hospital (approval number: 2016126). Written informed consent to participate was obtained from all subjects.

## Author contributions

HY and ZW designed the study and drafted the manuscript. YS, WB, KD, and CS collected the MRI data. YH and JZ analyzed and interpreted the results of the data. ZM and NW revised the manuscript. All authors approved the final manuscript.

## Footnotes

1
http://www.itk-snap.org

2
https://www.fil.ion.ucl.ac.uk/spm/

3
https://www.radiomics.io/pyradiomics.html

4
https://scikit-learn.org/
