# Supplementary material for: Prognosis of ischemic stroke predicted by machine learning based on multi-modal MRI radiomics
Source: Front Psychiatry. 2023 Jan 9;13:1105496. doi: 10.3389/fpsyt.2022.1105496 (PMC9868394; doi:10.3389/fpsyt.2022.1105496)
Supplement: Supplementary file 1 [file Data_Sheet_1.docx]

Supplementary Material

Prognosis of ischemic stroke predicted by machine learning based on multi-modal MRI radiomics

**Huan Yu^^[[1]](#footnote-2)^^, Zhenwei Wang^1^, Yiqing Sun^1^, Wenwei Bo^1^, Kai Duan^1^, Chunhua Song^1^, Yi Hu^1^,
Jie Zhou^1^, Zizhang Mu^2^, Ning Wu^3*^**

*** Correspondence:** Ning Wu: ning.wu@ccmu.edu.cn

# Definitions of radiomic features

First-order statistics describe the distribution of voxel intensities within the image region defined by the mask through commonly used and basic metrics.

Gray Level Run Length Matrix (glrlm) quantifies gray level runs, which are defined as the length in number of pixels, of consecutive pixels that have the same gray level value.

Gray Level Co-occurrence Matrix (glcm) describes the second-order joint probability function of an image region constrained by the mask.

Gray Level Dependence Matrix (gldm) quantifies gray level dependencies in an image.

Gray Level Size Zone Matrix (glszm) quantifies gray level zones in an image.

$\mathrm{MeshVolume}=\sum_{i=1}^{N_{f}} V_{i}$, is calculated from the triangle mesh of the ROI.

$\mathrm{LowGrayLevelRunEmphasis}=\frac{\sum_{i=1}^{N_{g}} \sum_{j=1}^{N_{r}} \frac{\text{P}(i,j|\theta)}{i^{2}}}{N_{r} (\theta)}$ , measures the distribution of low gray-level values, with a higher value indicating a greater concentration of low gray-level values in the image.

$\mathrm{ShortRunLowGrayLevelEmphasis}=\frac{\sum_{i=1}^{N_{g}} \sum_{j=1}^{N_{r}} \frac{\text{P}(i,j|\theta)}{i^{2}j^{2}}}{N_{r} (\theta)}$, measures the joint distribution of shorter run lengths with lower gray-level values.

$\text{IDN}=\sum_{k=0}^{N_{g}-1} \frac{p_{x-y} (k)}{1+(\frac{k}{N_{g}})}$, IDN (inverse difference normalized) is a measure of the local homogeneity of an image.

$\text{Maximum}=\max(\text{X})$, is the maximum gray level intensity within the ROI.

$\text{TotalEnergy}=V_{voxel}\sum_{i=1}^{N_{p}} {(\text{X}(i)+c)}^{2}$ , is the value of Energy feature scaled by the volume of the voxel in cubic mm.

$\mathrm{LargeDependenceHighGrayLevelEmphasis}=\frac{\sum_{i=1}^{N_{g}} \sum_{j=1}^{N_{d}} \text{P}(i,j)i^{2}j^{2}}{N_{z}}$ , measures the joint distribution of large dependence with higher gray-level values.

$\text{LargeAreaEmphasis}=\frac{\sum_{i=1}^{N_{g}} \sum_{j=1}^{N_{s}} \text{P}(i,j)j^{2}}{N_{z}}$ , a measure of the distribution of large area size zones, with a greater value indicative of more larger size zones and more coarse textures.

$\text{LargeAreaHighGrayLevelEmphasis}=\frac{\sum_{i=1}^{N_{g}} \sum_{j=1}^{N_{s}} \text{P}(i,j)i^{2}j^{2}}{N_{z}}$ , measures the proportion in the image of the joint distribution of larger size zones with higher gray-level values.

$\text{IDMN}=\sum_{k=0}^{N_{g}-1} \frac{p_{x-y}(k)}{1+(\frac{k^{2}}{N_{g}^{2}})}$ , a measure of the local homogeneity of an image.

$\text{IMC 1}=\frac{HXY-HXY1}{max\{HX,HY\}}$ , assesses the correlation between the probability distributions of i and j (quantifying the complexity of the texture).

$\text{IMC 2}=\sqrt{1-e^{-2(HXY2-HXY)}}$ , also assesses the correlation between the probability distributions of i and j (quantifying the complexity of the texture).

$\text{ZoneVariance}=\sum_{i=1}^{N_{g}} \sum_{j=1}^{N_{s}} p(i,j)(j-\mu)^{2}$ , measures the variance in zone size volumes for the zones.

$\text{LargeAreaLowGrayLevelEmphasis}=\frac{\sum_{i=1}^{N_{g}} \sum_{j=1}^{N_{s}} \frac{\text{P}(i,j)j^{2}}{i^{2}}}{N_{z}}$ , measures the proportion in the image of the joint distribution of larger size zones with lower gray-level values.

# Description of five machine learning models

SVM performs classification by constructing an N-dimensional hyper plane that optimally separates the data into two categories.

Random forest is a meta estimator that builds a number of randomized decision tree classifiers on various sub-samples of the dataset and uses averaging to improve the predictive accuracy and control over-fitting.

CatBoost, LightGBM, and XGBoost are all variations of gradient boosting algorithms.

In CatBoost, a greedy method is used such that a list of possible candidates of feature-split pairs are assigned to the leaf as the split and the split that results in the smallest penalty is selected.

In LightGBM, Gradient-based One-Side Sampling (GOSS) keeps all data instances with large gradients and performs random sampling for data instances with small gradients. Gradient refers to the slope of the tangent of the loss function.

In XGBoost, the pre-sorted algorithm considers all feature and sorts them by feature value. After which, a linear scan is done to decide the best split for the feature and feature value that results in the most information gain.

1. [↑](#footnote-ref-2)
